# Patients’ access to rare neuromuscular disease therapies varies across US private insurers

**DOI:** 10.1186/s13023-022-02182-3

**Published:** 2022-02-05

**Authors:** Nikoletta M. Margaretos, Komal Bawa, Natalie J. Engmann, James D. Chambers

**Affiliations:** 1 Center for the Evaluation of Value and Risk in Health, Tufts Medical Center, Boston, MA USA; 2 Genentech, Inc., South San Francisco, CA USA

**Keywords:** Patient access, Managed care, Rare disease, Neuromuscular disease

## Abstract

**Background:**

The extent to which different US private insurers require their enrollees to meet the same coverage criteria before gaining access to treatment is unclear. Our objective was to scrutinize the patient access criteria imposed by US private insurers for a set of rare neuromuscular disease (NMD) disease-modifying therapies (DMTs).

**Methods:**

We examined coverage policies issued by 17 large US private insurers for the following NMD treatments: nusinersen and onasemnogene abeparvovec for spinal muscular atrophy, edaravone for amyotrophic lateral sclerosis, and eteplirsen for Duchenne muscular dystrophy. We reviewed the plans’ coverage policies and identified the patient access criteria, including clinical prerequisites, step therapy protocols, and prescriber requirements. We compared the plans’ patient access criteria with the therapies’ US Food and Drug Administration (FDA)-labeled indications.

**Results:**

The included insurers issued 65 coverage policies for the included therapies. Plans imposed coverage restrictions beyond the FDA-approved indications in 60 coverage policies; plans did not cover eteplirsen in five policies. No therapy was covered the same way by all insurers. Plans applied clinical criteria beyond the FDA label indication in 56 policies and step therapy protocols in three policies. Plans required that a neurologist prescribe the therapy in 37 policies, 22 of which required the neurologist to have expertise in the particular disease. Plans often required patients to suffer from symptoms of particular severity; e.g. for eteplirsen, plans differed in their 6-min walk test requirements; for edaravone, some plans required that patients had normal respiratory function, while others required only that patients did not require ventilation; for nusinersen and onasemnogene abeparvovec, plans differed in the number of *SMN2* gene copies they required patients to have (*SMN2* copy number is correlated with disease severity).

**Conclusions:**

The evaluated large US private insurers tended to impose coverage restrictions beyond the FDA label indication for the included set of rare NMD DMTs. Plans rarely applied the same patient access criteria in their coverage policies for the same products. Inconsistent coverage criteria mean that patients with different insurers have variable access to the same therapies across insurers.

**Supplementary Information:**

The online version contains supplementary material available at 10.1186/s13023-022-02182-3.

## Background

The high cost of providing coverage for rare disease therapies remains a key challenge for health insurers. This challenge will increase as regulatory agencies continue to approve increasingly large numbers of rare disease therapies and healthcare payers have to balance providing access with budget constraints [1, 2].

Unlike healthcare systems in most other developed countries, the US healthcare system consists of a complex set of public (e.g. Medicare and Medicaid) and private insurers [3]. Roughly half of Americans receive health insurance through one of the many private insurers. A consequence of the US healthcare system’s complexity is the variation in insurers’ drug coverage policies, which can lead to patients having inconsistent access to care and potentially create discontinuities in drug therapy for patients moving from one health plan to another [4].

Research has found that while private insurers are more likely to cover drugs for rare diseases than more prevalent diseases, they nevertheless impose coverage criteria that go beyond the US Food and Drug Administration (FDA)-labeled indication roughly 30% of the time (compared with almost 50% of the time for non-orphan drugs) [5]. Research has shown that private insurers vary in how often they impose restrictions in their orphan drug coverage decisions; i.e. some plans impose coverage restrictions more frequently than other plans [3]. Insurers typically restrict patients’ access to orphan drugs by imposing additional clinical requirements, e.g. requiring patients to present with a particular set of symptoms or have disease of particular severity or duration. However, whether plans apply the same clinical requirements, i.e. require patients to meet the same clinical criteria before gaining access to treatment, is unknown.

In this study, we address this gap in our understanding by examining the clinical criteria that US private insurers impose in their coverage policies for a set of rare neuromuscular disease (NMD) disease-modifying therapies (DMTs): nusinersen and onasemnogene abeparvovec-xioi for spinal muscular atrophy (SMA), edaravone for amyotrophic lateral sclerosis (ALS), and eteplirsen for Duchenne muscular dystrophy (DMD) (Table 1). In contrast to treatments that treat a patient’s symptoms, DMTs slow disease progression by altering the underlying disease. We focus on these NMDs, as new therapeutics in these diseases have recently received FDA approval when previously either no or limited therapeutic options were available, and the frequency with which private insurers issued coverage policies for them.

**Table 1:** FDA-approved indications for included treatments

International nonproprietary name	Brand name	FDA-approved indication
Onasemnogene abeparvovec—xioi	Zolgensma	ZOLGENSMA (onasemnogene abeparvovec-xioi) is an adeno-associated virus vector-based gene therapy indicated for the treatment of pediatric patients less than 2 years of age with spinal muscular atrophy (SMA) with bi-allelic mutations in the *survival motor neuron 1* (*SMN1*) gene *Limitation of Use* The safety and effectiveness of repeat administration of ZOLGENSMA have not been evaluated The use of ZOLGENSMA in patients with advanced SMA (e.g., complete paralysis of limbs, permanent ventilator dependence) has not been evaluated
Nusinersen	Spinraza	SPINRAZA is a *survival motor neuron-2* (*SMN2*)-directed antisense oligonucleotide indicated for the treatment of spinal muscular atrophy (SMA) in pediatric and adult patients
Edaravone	Radicava	RADICAVA is indicated for the treatment of amyotrophic lateral sclerosis (ALS)
Eteplirsen	Exondys 51	EXONDYS 51 is an antisense oligonucleotide indicated for the treatment of Duchenne muscular dystrophy (DMD) in patients who have a confirmed mutation of the DMD gene that is amenable to exon 51 skipping. This indication is approved under accelerated approval based on an increase in dystrophin in skeletal muscle observed in some patients treated with EXONDYS 51 [see Clinical Studies (14)] A clinical benefit of EXONDYS 51 has not been established. Continued approval for this indication may be contingent upon verification of a clinical benefit in confirmatory trials

*Source* Drugs@FDA: FDA-Approved Drugs (https://www.accessdata.fda.gov/scripts/cder/daf/)

*FDA* US Food and Drug Administration

SMA is a genetic disorder that manifests due to deletions and/or mutations in the *survival motor neuron* (*SMN) 1* gene resulting in progressive muscle weakness and atrophy [6]. There are four types of clinical SMA, of which Type 1 is the most severe. In Type 1 SMA, an infant’s respiratory function is impaired, and life expectancy without treatment, based on natural history, is typically less than 2 years. Disease severity is associated with the number of *SMN2* gene copies, with three or more copies associated with less severe disease. In 2016, the FDA approved nusinersen, the first drug to treat children and adults with SMA [7]. Nusinersen is an *SMN2*-targeting antisense oligonucleotide, which increases the ability of cells to produce functional SMN protein [8]. In 2019, the FDA approved onasemnogene abeparvovec for the treatment of children younger than 2 years of age with SMA. Onasemnogene abeparvovec is a gene therapy that works by introducing a new copy of the gene that makes the SMN protein [9].

ALS is a progressive neurodegenerative disease that results in loss of upper and lower motor neurons, with an onset between 40 and 70 years of age (average age 55) [10, 11]. Initial presentation typically involves limb weakness or difficulty with swallowing and speech. Roughly 90% of ALS cases are ‘sporadic’, meaning it occurs in patients without a family history; 10% are ‘familial’ and are inherited through a mutated gene [12]. While the prognosis is variable, life expectancy averages 2–5 years from time of diagnosis due to respiratory paralysis [9]. The FDA approved edaravone to treat patients with ALS in 2017, making it only the second ALS treatment approved by the FDA since the agency approved riluzole in 1995 [13, 14]. While edaravone’s exact mechanism of action is unclear, it is thought to work by reducing the effects of oxidative stress, which contributes to the process that kills neurons in patients with ALS [15].

DMD is an inherited disorder caused by loss-of-function mutations of the *dystrophin* gene, which is required for the protein product necessary to maintain muscle cell integrity [16]. The disease typically manifests in boys between the ages of 3 and 5 years. Symptoms are caused by progressive muscle wasting that results in difficulty in ambulation before age 12 and cardiomyopathy in the teenage years, with a life expectancy of around 30 years with current approved therapies and disease management. In 2016, the FDA approved eteplirsen, the first treatment for patients with DMD [17]. Eteplirsen binds to exon 51 of the *DMD* gene, causing the body to ‘skip’ it from the mature mRNA transcript. In so doing, the drug allows the successful translation of a shortened, but functional, dystrophin protein [18].

This study is the first to scrutinize the patient access criteria imposed by private insurers for therapies for these diseases.

## Methods

We used the Tufts Medical Center Specialty Drug Evidence and Coverage (SPEC) Database for this research. The SPEC Database includes information that researchers manually extract from the publicly available coverage policies that health plans post on their websites. The SPEC Database includes information on how 17 of the 20 largest US private insurers (in terms of premiums earned) cover over 290 specialty drugs and products (see Additional file 1) [4]. Of the three excluded plans, two focus exclusively on public payers (Medicare or Medicaid populations), and one does not make its coverage decisions publicly available. SPEC includes 6 national and 11 regional private insurers. The insurers in SPEC represent roughly 150 million covered lives, which is approximately 60% of the private health insurance market. The SPEC Database is updated three times each year. Coverage information included in this study was current through April 2020.

The SPEC Database contains information on how the included plans cover specialty products for their enrollees, i.e. detail on the clinical criteria that insurers apply in their coverage policies. For instance, it captures any coverage requirements related to a patient’s genetic presentation, disease severity, and any step therapy protocol requirements, i.e. treatment failure on a specific therapy before access to an alternative treatment.

We examined coverage policies in SPEC for the following treatments: nusinersen and onasemnogene abeparvovec for SMA (a gene therapy), edaravone for ALS, and eteplirsen for DMD. At the time of the analysis, these were the only specialty products FDA approved for these diseases. We reviewed coverage policies that the plans issued for these therapies and identified the specific coverage criteria required by each plan beyond the FDA label indication, including clinical criteria prerequisites, step therapy protocols, and prescriber requirements. We compared and contrasted the coverage criteria required by the included insurers.

## Results

The included insurers issued 65 (out of a total possible 68) coverage policies for these rare NMD therapies. All 17 plans issued a coverage policy for nusinersen; 16 plans issued a policy for onasemnogene abeparvovec, eteplirsen, and edaravone.

### Onasemnogene abeparvovec for SMA

All 16 insurers that issued a coverage policy for onasemnogene abeparvovec applied conditions on coverage beyond the FDA label (Table 2). All plans required patients to have a confirmed diagnosis of SMA based on genetic criteria, e.g. bi-allelic *SMN1* loss-of-function gene mutations. However, plans varied in their specific genetic requirements. Four plans reserved coverage for patients with Type 1 SMA and two plans covered Type 1 or 2 SMA, while ten plans did not address SMA type in their coverage policies (the FDA label did not specify type of SMA). Plans were inconsistent in the number of *SMN2* gene copies required.

**Table 2:** Payer coverage criteria for onasemnogene abeparvovec for spinal muscular atrophy

	Plan imposes additional coverage criteria	Covered types of SMA	Required number of copies of *SMN2* gene	Age requirement	Coverage for advanced SMA*	Prescriber requirement
Plan 1	Yes	NA	NA	< 2 years	No	Neurologist with expertise in SMA
Plan 2	Yes	1 only	1 or 2 copies	< 2 years	NA	NA
Plan 3	Yes	NA	1, 2, or 3 copies	< 2 years	No	Neurologist
Plan 4	Yes	NA	1, 2, or 3 copies	< 2 years	No	Neurologist with expertise in SMA
Plan 5	Yes	NA	1, 2, or 3 copies	< 2 years	No	Neurologist with expertise in SMA
Plan 6	Yes	NA	1, 2, or 3 copies	< 2 years	No	Neurologist with expertise in SMA
Plan 7	Yes	1 only	1, 2, or 3 copies	< 2 years	No	Neurologist with expertise in SMA
Plan 8	Yes	NA	1, 2, or 3 copies	< 2 years	No	NA
Plan 9	Yes	1 only	2 copies	< 9 months	No	Neurologist with expertise in SMA
Plan 10	Yes	1 only	1, 2, or 3 copies	< 2 years	No	Neurologist
Plan 11	Yes	NA	1, 2, or 3 copies	< 2 years	No	Neurologist with expertise in SMA
Plan 12	Yes	1 or 2	1, 2, or 3 copies	< 2 years	No	Neurologist with expertise in SMA
Plan 13	No policy	No policy	No policy	No policy	No policy	No policy
Plan 14	Yes	NA	2 or 3 copies	< 2 years	NA	NA
Plan 15	Yes	NA	1, 2, or 3 copies	< 2 years	No	Neurologist
Plan 16	Yes	NA	1 or 2 copies	< 2 years	No	Neurologist with expertise in SMA
Plan 17	Yes	1 or 2	1, 2, or 3 copies	< 2 years	No	Neurologist with expertise in SMA

NA = Payer did not address criterion in their coverage policy; *Insurers typically define advanced SMA as patients with complete paralysis of limbs, or on permanent ventilator dependence

*NA* not available, *SMA* spinal muscular atrophy, *SMN* survival motor neuron

Plans typically required that patients be less than 2 years of age at the time of infusion, although one plan required the patient to be less than 9 months of age. Fourteen plans did not cover onasemnogene abeparvovec for patients with advanced SMA, which was typically defined as patients with complete paralysis of their limbs, and/or on permanent ventilation. Two insurers did not address stage of disease in their coverage policies.

Thirteen plans required that a neurologist prescribe onasemnogene abeparvovec; ten of these plans stipulated that the neurologist had specific expertise in managing patients with SMA.

### Nusinersen for SMA

All 17 plans that issued a coverage policy for nusinersen applied conditions on coverage beyond the FDA label (Table 3). Similar to onasemnogene abeparvovec, all plans required patients to have a confirmed diagnosis of SMA based on genetic criteria, e.g. either homozygous deletion or dysfunctional mutation of the *SMN1* gene. However, plans varied with respect to their specific genetic testing results requirements. Thirteen plans explicitly covered the drug for patients with Type 1, 2, or 3 SMA, whereas four plans did not address SMA type in their coverage policy. Eight plans required patients to have a specific number of *SMN2* gene copies but differed with respect to the number of copies required for coverage.

**Table 3:** Payer coverage criteria for nusinersen for spinal muscular atrophy

	Plan imposes additional coverage criteria	Covered types of SMA	Required number of copies of *SMN2* gene	Age requirement at treatment initiation	Ventilation requirements*	Motor function requirements	Prescriber requirement
Plan 1	Yes	1, 2, or 3	NA	≤ 15 years	Patient is not ventilator dependent*	NA	Neurologist with expertise in SMA
Plan 2	Yes	NA	1 or 2 copies	NA	NA	NA	NA
Plan 3	Yes	1, 2, or 3	NA	NA	NA	NA	Neurologist
Plan 4	Yes	NA	NA	NA	NA	NA	Neurologist
Plan 5	Yes	1, 2, or 3	NA	≤ 14 years	Patient is not ventilator dependent*	NA	Neurologist with expertise in SMA
Plan 6	Yes	NA	≥ 2 copies	NA	NA	NA	Neurologist
Plan 7	Yes	1, 2, or 3	NA	NA	NA	Member retains meaningful voluntary motor function^†^	NA
Plan 8	Yes	1, 2, or 3	1, 2, or 3 copies	NA	NA	Member retains meaningful voluntary motor function^†^	NA
Plan 9	Yes	1, 2, or 3	NA	NA	Not dependent for > 6 h a day	NA	Neurologist with expertise in SMA
Plan 10	Yes	1, 2, or 3	1, 2, 3, or 4 copies	NA	Patient is not ventilator dependent*	NA	NA
Plan 11	Yes	1, 2, or 3	NA	≤ 15 years	Patient is not ventilator dependent*	Member retains meaningful voluntary motor function^†^	Neurologist
Plan 12	Yes	1, 2, or 3	1 or 2 copies	≤ 15 years	Not dependent for > 12 h a day	NA	Neurologist with expertise in SMA
Plan 13	No policy	1, 2, or 3	NA	≤ 15 years	NA	NA	NA
Plan 14	Yes	1, 2, or 3	Symptomatic patients: 2, 3, or 4 copies; Asymptomatic patients: 2 or 3 copies	≤ 15 years	Patient is not ventilator dependent*	NA	Neurologist with expertise in SMA
Plan 15	Yes	NA	Early onset: 1 or 2 copies; Late onset: 1, 2, or 3 copies	NA	NA	NA	NA
Plan 16	Yes	1, 2, or 3	NA	NA	NA	NA	Neurologist
Plan 17	Yes	1, 2, or 3	≥ 2 copies	NA	Patient is not ventilator dependent*	NA	Neurologist with expertise in SMA

NA = Payer did not address criterion in their coverage policy; *Permanent ventilation (defined as tracheostomy or ventilatory support for at least 16 h per day for more than 21 continuous days in the absence of an acute reversible event); ^†^Meaningful motor function typically defined as the ability to manipulate objects using upper extremities, walk, etc.

*NA* not available, *SMA* spinal muscular atrophy, *SMN* survival motor neuron

Six plans required that treatment be initiated before a certain age; five plans required 15 years of age or younger, whereas one plan required 14 years of age or younger. Eight plans had coverage requirements with respect to ventilation; however, the definition of ventilator dependence varied among these plans. Three plans required that the patient had meaningful motor function (e.g. ability to walk or to manipulate objects using upper extremities).

Eleven plans required that a neurologist prescribe nusinersen; six of these plans stipulated that the physician had specific expertise in managing patients with SMA.

### Edaravone for ALS

All 16 insurers that issued a coverage policy for edaravone applied conditions on coverage beyond the FDA label (Table 4). All plans required that patients had a diagnosis of ALS, but specific requirements differed. For instance, 11 plans required a definite or probable diagnosis based on El Escorial revised criteria, whereas five plans did not. Thirteen plans required patients to have disease duration of less than 2 years, whereas three plans did not include disease duration as a criterion in their coverage policy.

**Table 4:** Payer coverage criteria for edaravone for amyotrophic lateral sclerosis

	Plan imposes additional coverage criteria	Japan ALS severity classification grade < 3	≥ 2 points or better on each item of the ALSFRS-R*	Respiratory status^†^	Use of El Escorial revised criteria for diagnosis	Disease duration of ≤ 2 years	Prior therapy with riluzole	Prescriber requirement
Plan 1	Yes	No	Yes	Ventilation is not required	No	NA	NA	NA
Plan 2	Yes	Yes	Yes	Normal respiratory function	Yes	Yes	NA	NA
Plan 3	Yes	No	Yes	NA	Yes	Yes	NA	Neurologist
Plan 4	No policy	No policy	No policy	No policy	No policy	No policy	No policy	No policy
Plan 5	Yes	No	No	Normal respiratory function	No	Yes	Yes	Neurologist
Plan 6	Yes	No	Yes	Normal respiratory function	No	Yes	NA	NA
Plan 7	Yes	No	Yes	Normal respiratory function	Yes	Yes	Yes	Neurologist with expertise in ALS
Plan 8	Yes	No	Yes	Normal respiratory function	Yes	Yes	NA	NA
Plan 9	Yes	No	Yes	Ventilation is not required	No	NA	NA	NA
Plan 10	Yes	No	Yes	Normal respiratory function	Yes	Yes	NA	Neurologist
Plan 11	Yes	No	Yes	Normal respiratory function	Yes	Yes	NA	Neurologist
Plan 12	Yes	No	No	NA	No	Yes	Yes	Neurologist with expertise in ALS
Plan 13	Yes	No	Yes	Normal respiratory function	Yes	Yes	NA	NA
Plan 14	Yes	No	Yes	Normal respiratory function	Yes	Yes	NA	NA
Plan 15	Yes	No	Yes	NA	Yes	Yes	NA	NA
Plan 16	Yes	No	Yes	Normal respiratory function	Yes	Yes	NA	Neurologist
Plan 17	Yes	No	Yes	Normal respiratory function	Yes	NA	NA	Neurologist with expertise in ALS

NA = Payer did not address criterion in their coverage policy; *A measure of patient functioning, ALSFRS-R = ALS Functional Rating Scale-Revised; ^†^Normal respiratory function (defined as percent predicted forced vital capacity values of ≥ 80%)

*ALS* amyotrophic lateral sclerosis, *NA* not available

Fourteen plans required that patients had the ability to perform most activities of daily living (defined as scores of 2 points or better on each individual item of the ALS Functional Rating Scale—Revised). Two plans did not include a coverage criterion related to patient function. One plan additionally required that the patient have a Japan ALS severity classification grade of less than 3 at the time of therapy initiation. Eleven plans required that patients had normal respiratory function (defined as percent predicted forced vital capacity values of ≥ 80%). In contrast, two plans required that the patient was not ventilator dependent.

Three plans required that patients first try riluzole and experience treatment failure before being prescribed edaravone. Eight plans required that a neurologist prescribe edaravone; three of these plans stipulated that the physician had specific expertise in managing patients with ALS.

### Eteplirsen for DMD

Sixteen insurers issued a coverage policy for eteplirsen (Table 5). Five plans did not cover eteplirsen for their enrollees, as they considered the therapy to be experimental/investigational (not medically necessary). Eleven plans covered the therapy for their enrollees and applied additional coverage criteria beyond the FDA label.

**Table 5:** Payer coverage criteria for eteplirsen for Duchenne muscular dystrophy

	Plan covers therapy?	Plan imposes additional coverage criteria	6-min walk test (meters)	Ambulation/motor function	Age requirement	Prior therapy with glucocorticoids	Prescriber requirement
Plan 1	Yes	Yes	≥ 180 m	NA	Therapy initiated < 14 years of age	NA	Neurologist with expertise in DMD
Plan 2	Yes	Yes	NA	Ambulation with or without assistance	NA	NA	NA
Plan 3	Yes	Yes	≥ 300 m	Ambulation with or without assistance	NA	NA	Neurologist
Plan 4	Yes	Yes	NA	Ambulation without assistance	NA	NA	Neurologist
Plan 5	No	NC	NC	NC	NC	NC	NC
Plan 6	No	NC	NC	NC	NC	NC	NC
Plan 7	No	NC	NC	NC	NC	NC	NC
Plan 8	Yes	Yes	NA	Voluntary motor function (e.g. ambulate, able to speak, manipulate objects)	NA	Yes	NA
Plan 9	Yes	Yes	≥ 180 m	NA	Therapy initiated < 14 years of age	NA	NA
Plan 10	Yes	Yes	≥ 200 m	Ambulation with or without assistance	Therapy initiated < 13 years of age	Yes	Neurologist
Plan 11	Yes	Yes	NA	Ambulation with or without assistance	NA	NA	NA
Plan 12	Yes	Yes	≥ 300 m	Ambulation without assistance	Patient is ≥ 7 years	Yes	Neurologist with expertise in DMD
Plan 13	No	NC	NC	NC	NC	NC	NC
Plan 14	No	NC	NC	NC	NC	NC	NC
Plan 15	Yes	Yes	NA	Ambulation with or without assistance	NA	NA	NA
Plan 16	No policy	No policy	No policy	No policy	No policy	No policy	No policy
Plan 17	Yes	Yes	≥ 300 m	Ambulation without assistance	NA	NA	Neurologist with expertise in DMD

NA = Payer did not address criterion in their coverage policy; NC = The plan did not cover the therapy for their enrollees

*DMD* Duchenne muscular dystrophy, *NA* not available, *NC* not covered

Four plans included an age requirement in their coverage policy. Two plans required the patient to be aged 14 years or less at therapy initiation, one plan required the patient to be aged 13 years or less at therapy initiation, and one plan required the patient to be aged 7 years or more to be eligible for treatment. All plans required that patients were able to ambulate, yet how this stipulation was met varied among the plans. For instance, five plans required that patients were ambulatory with or without an assistive device (e.g. a cane or a walker), whereas three plans required that the patient be ambulatory *without* an assistive device.

Three plans required that patients had been adherent to glucocorticoid therapy before receiving eteplirsen. Six plans required that a neurologist prescribe eteplirsen; three of these plans stipulated that the physician had specific expertise in managing patients with DMD.

## Discussion

In this study, we scrutinized the clinical criteria that large private insurers impose in their coverage policies for a set of rare NMD DMTs. We found that while plans typically covered the drugs for their enrollees, they tended to apply coverage criteria beyond the FDA label. Notably, we found that different plans rarely applied the same criteria in their coverage policies, which is a finding that is consistent with previous research [5]. This variation can have important consequences for patients’ access to care, as patients with different insurers can have different access to the same therapies. Furthermore, this variation could potentially result in loss of access to a therapy for patients moving from one plan to another plan that has different drug coverage criteria. The differences in coverage criteria are often subtle and are likely intended for utilization management, but can create confusion or frustration for patients and their families in an already complex healthcare system. For physicians, differences in coverage criteria can make assisting their patients with access to treatment difficult and mean that treatment decisions must be tailored not only to the patient’s clinical presentation but also the patient’s insurance coverage. For instance, for nusinersen, of the insurers that stipulated an age requirement for treatment, one of the six plans differed with respect to the eligibility age. Particularly for diseases with a high burden of illness, delays in access to therapy due to lengthy appeals processes may negatively impact both patients and caregivers [19, 20].

Our study focused on 17 of the largest US commercial plans. Future research should examine whether similar variation is observed among the coverage policies for NMD DMTs issued by smaller commercial insurers. Research that compares how public (e.g. Medicaid) and commercial plans cover NMD DMTs would also be valuable.

Insurers often look to products’ Phase 3 clinical trials when formulating their drug coverage criteria. However, the identified differences in insurers’ coverage criteria often related to aspects of disease severity not directly related to the clinical trial inclusion criteria, which may suggest that in some cases, these criteria were somewhat subjective. For example, the plans that covered eteplirsen for DMD differed with respect to the degree of required ambulation. Plans varied in the required 6-min walk distance, and though some plans permitted a patient to be ambulatory with or without assistance, other plans required the patient to be ambulatory without assistive devices. For edaravone, some plans required that patients had normal respiratory function, while others required only that patients did not require ventilation. Similarly, for nusinersen, of the eight plans that noted ventilator dependence, the definition of ‘dependence’ varied substantially from less than 6 h/day to less than 16 h/day.

It was notable that for the two included SMA DMTs, the eligibility criteria varied with respect to the patient’s genetic presentation. Not all plans included specific coverage requirements with respect to the number of *SMN2* gene copies, and the requirements varied for those that did. Some plans tended to restrict coverage to patients with fewer *SMN2* copies (and thus more severe disease), whereas others covered for patients with more copies (thus covering the product for patients with less severe disease). Interestingly, two plans did not cover onasemnogene abeparvovec for patients with a single *SMN2* gene copy (most severe disease), whereas three plans did not cover the therapy for patients with three *SMN2* gene copies (less severe disease). We identified a parallel finding for nusinersen for SMA, for which three plans did not cover the therapy for patients with a single *SMN2* gene copy, whereas two plans did not cover the product for patients with three or four *SMN2* gene copies.

We found that plans often required that a specialist, or a specialist with particular expertise, prescribe the drug, although plans did not define what level of expertise was necessary. Such requirements may be necessary when specialized training is required for the safe and effective use of a drug. However, there is a risk that prescriber requirements delay patients’ access to care, make care more expensive, and create access challenges, particularly for some rurally located patients and those without access to physicians with the required expertise [21].

Insurers face a challenge to accommodate the increasing number of costly drugs for rare diseases, such as those included in this study. Subsequent to the implementation of the Orphan Drug Act [22], research has found that the FDA often imposes a different evidence standard for drugs studied in rare diseases compared with those for more prevalent diseases (e.g. smaller sample sizes, lack of randomization) [5]. In addition, rare diseases pose unique challenges to evidence generation, both for clinical trial enrollment and real-world evidence (RWE) generation, leading to less robust data across the spectrum of patients with the disease. Extrapolating the results of clinical studies to all patients with a particular disease is difficult, leaving insurers to discern how to cover these treatments for their beneficiaries. Indeed, despite eteplirsen receiving FDA approval, some insurers did not cover the treatment, with their rationale being that evidence of clinical efficacy was inconclusive.

Importantly, insurers in our sample covered the products for a narrower patient population than the FDA label indication. For instance, while the FDA approved edaravone, ‘*for the treatment of amyotrophic lateral sclerosis*’, plans often followed the more precise clinical study inclusion criteria (as reported in the product’s label) in their coverage policies [13]. In these policies, plans required the following clinical study inclusion criteria: (1) functionality retained as defined by scores of 2 points or better on each individual item of the ALS Functional Rating Scale—Revised; (2) normal respiratory function; (3) definite or probable ALS based on El Escorial revised criteria; and (4) disease duration of 2 years or less. Overall, half of the plans that issued a decision for edaravone embedded each of these criteria in their coverage policy, and the remaining included some of these criteria.

In other cases, the plans did not adhere to the registration study’s inclusion criteria so closely. For example, patients included in the clinical study reported in onasemnogene abeparvovec’s FDA label had two *SMN2* gene copies [9]. However, as noted above, only one plan required patients to have exactly two *SMN2* gene copies, whereas ten plans permitted access to patients with one, two, or three *SMN2* gene copies.

Given the limited supporting evidence and relatively high cost of a rare disease therapy, it is understandable that decision makers would look to the products’ pivotal trial inclusion criteria when formulating their coverage decisions for novel drugs for rare diseases. However, reflecting these inclusion criteria in a product’s coverage policy can be problematic [23]. For example, clinical measures and scales are typically administered and assessed in a controlled clinical trial setting and may not be practical for use in an outpatient setting. A plan’s incorporation of these measures and scales in initial approval or recertification criteria can put undue burden on the prescribing physician and the patient, adding to the complexity of patient management. Moreover, the prescribing physician must be able to correctly administer these scales, which can be complex and time-consuming. Furthermore, rigidly adhering to clinical trial measures and scales to guide patient access likely results in patients’ circumstances and preferences not being included. For example, the Hammersmith Functional Motor Scale – Expanded (HFMSE) is typically administered in patients with SMA who are able to sit or walk [24]. A 3-point increase in HFMSE score is thought to represent the minimum change considered ‘clinically meaningful’, while a 1-point change may be considered meaningful to caregivers of non-ambulatory patients with Type 2 SMA [25]. Additionally, stabilization of disease, noted through no change or decrease on the scale, is valuable in degenerative diseases. This is one example of the many scales administered in clinical trials depending on the type of SMA, disease burden, and the patient’s age [26].

That some plans adhere more closely to the clinical trial inclusion criteria than other plans may explain some of the variation in plan decision-making. However, plans may differ in their coverage decisions for various other reasons that are not shown in publicly available information. Plans likely have different available budgets for rare diseases, leading some to cover products more generously than others. Plan contracting with product manufacturers may also influence how coverage of different therapies is prioritized. Differences in the evidence that plans review when formulating their decisions may also affect how they cover therapies. Publications have established that the evidence plans report reviewing in their coverage policies varies, including the frequency that they review RWE [27, 28].

Our study highlights the importance of continued evidence generation in real-world settings. RWE can help to address the limitations of the clinical studies that the FDA used for drug approval. By evaluating how a drug performs in patients excluded from trials, RWE can help to determine how best to use therapies in clinical practice. For rare diseases, patient registries are now a common source of RWE. Various registries for NMDs exist, including NeuroMuscular ObserVational Research (MOVR) data, the International SMA Consortium (iSMAC), Duchenne Registry, and National ALS Registry [29–31]. However, while registries can be a valuable source of RWE, the time needed for the collection of sufficient and robust RWE can be problematic for broader patient access, particularly for recently approved therapies.

Despite the limited evidence supporting many drugs for rare diseases, healthcare decision makers must find a way to balance appropriate patient access while mitigating high treatment costs. A promising approach is to use an outcomes-based agreement, which ties payment for drugs to positive health outcomes for patients. An outcomes-based agreement allows an insurer to provide patients access to a therapy while reducing the risk of paying for costly treatment with uncertain benefits [32]. However, employing these agreements in practice is restricted by high implementation costs, data infrastructure requirements, and measurement challenges [33]. To date, the success of outcomes-based agreements has been unclear [34]. For therapies with high upfront treatment costs, such as one-dose gene therapies, an approach that can be used in conjunction with outcomes-based agreements is to spread the cost of the treatment (both for the insurer and the patient) over the period that a patient experiences positive health outcomes, which can mitigate the affordability challenge [35].

Our study has a number of limitations. First, the four included therapies may not be generalizable to other therapies indicated for NMDs, or to orphan diseases more generally. Our findings may also not be generalizable to other private insurers or to public healthcare payers (e.g. Medicaid). Secondly, when a plan does not issue a coverage policy for a therapy, we do not know what access the plan provides to its enrollees. In these circumstances, the plan may have an internal coverage policy, or the plan may adjudicate coverage on a case-by-case basis. Thirdly, we do not account for the appeals process that insurers provide to patients for denied coverage claims. Fourthly, we do not examine whether the additional clinical coverage criteria are noted in the individual clinical trials. Lastly, as our analysis was conducted in April 2020, we do not include DMTs indicated for the considered NMDs that were approved by the FDA after that date. For example, risdiplam was approved by the FDA in August 2020 and, therefore, was not included in our study.

## Conclusions

The evaluated set of large US private insurers tended to apply coverage restrictions beyond the FDA label indication in their coverage policies for a set of rare NMD DMTs. Plans rarely applied the same criteria in their coverage policies for the same products. Inconsistent coverage criteria mean that patients with different insurers have variable access to the same therapies, which may have important consequences for patients who move from one plan to another.

## Supplementary Information

**Additional file 1**: Private insurers included in the Specialty Drug Evidence and Coverage (SPEC) database.

## Data Availability

This analysis was based upon publicly available data. The data sets used and/or analyzed during the current study are available from the corresponding author on reasonable request.

## Abbreviations

ALS: Amyotrophic lateral sclerosis
DMD: Duchenne muscular dystrophy
DMT: Disease-modifying therapy
FDA: US Food and Drug Administration
HFMSE: Hammersmith Functional Motor Scale – Expanded
iSMAC: International SMA Consortium
MOVR: NeuroMuscular ObserVational research
NMD: Neuromuscular disease
RWE: Real-world evidence
SMA: Spinal muscular atrophy
SMN: Survival motor neuron
SPEC: Specialty Drug Evidence and Coverage
