# Supplementary material for: Patients’ access to rare neuromuscular disease therapies varies across US private insurers
Source: Orphanet J Rare Dis. 2022 Feb 5;17:36. doi: 10.1186/s13023-022-02182-3 (PMC8817582; doi:10.1186/s13023-022-02182-3)
Supplement: Supplementary file 1 — Additional file 1: Private insurers included in the Specialty Drug Evidence and Coverage (SPEC) database. [file 13023_2022_2182_MOESM1_ESM.docx]

**Online Appendix**

Commercial health plans included in the Specialty Drug Evidence and Coverage (SPEC) database

1. Aetna Inc.
2. Anthem Inc.
3. Blue Cross Blue Shield Massachusetts
4. Blue Cross Blue Shield Michigan
5. Blue Cross Blue Shield New Jersey
6. Blue Cross Blue Shield North Carolina
7. Blue Cross Blue Shield Tennessee
8. Carefirst Inc.
9. Centene Corporation
10. Cigna
11. EmblemHealth
12. Guidewell (Florida's Blue Cross and Blue Shield)
13. Health Care Service Corporation
14. Highmark Inc.
15. Humana Inc.
16. Independence Health Group
17. UnitedHealth Group

**Supplementary Table 1.**

Note: Payers listed in alphabetical order. Three of the largest 20 payers were excluded because they only offered Medicare/Medicaid coverage (n=2) or did not make their policies publicly available (n=1).

Source: 2019 Accident and Health Policy Experience Report. Market Share Reports For the Top 125 Accident and Health Insurance Groups and Companies by State and Countrywide. Page 253. Available here https://www.naic.org/prod_serv/AHP-LR-20.pdf.
